# Novel Soliton and Periodic Wave Solutions of the (3+1)-Dimensional Shallow Water Wave Equation with Bifurcation Analysis

**DOI:** 10.1038/s41598-025-21052-z

**Published:** 2025-10-20

**Authors:** Wafy M. Hasan, Hamdy M. Ahmed, Ahmed M. Ahmed, Haytham M. Rezk, Wafaa B. Rabie

**Affiliations:** 1 Department of Mathematics, Faculty of Science, Al-Azhar University, Cairo, Egypt; 2 Department of Basic Sciences, Faculty of Engineering Technology, ElSewedy University of Technology, Cairo, Egypt; 3 Department of Physics and Engineering Mathematics, Higher Institute of Engineering, El Shorouk Academy, Cairo, Egypt; 4 Department of Mathematics, Faculty of Science, Luxor University, Taiba, Luxor, Egypt

**Keywords:** Analytical solutions, Nonlinear wave interactions, High-order dispersion effects, Dynamical system bifurcation., Engineering, Mathematics and computing, Ocean sciences, Physics

## Abstract

This study derives novel exact traveling wave solutions for the $(3 + 1)-$dimensional shallow water wave equation–a pivotal model in coastal hydrodynamics for tsunami prediction and tidal analysis. By employing an enhanced tanh-function method, we obtain a diverse spectrum of solutions, including dark, singular, and periodic solitons, as well as hyperbolic, Jacobi elliptic, rational, and exponential forms, which surpass the variety and generality reported in previous studies. These solutions uncover previously unexplored wave propagation patterns and interaction dynamics. A comprehensive bifurcation analysis elucidates the stability and phase transitions of the wave solutions, providing deeper analytical insight into their behavior. High-resolution graphical visualizations quantitatively demonstrate wave amplification and nonlinear interactions, confirming the superiority of our method in capturing complex physical phenomena. The results not only advance nonlinear wave theory but also enhance predictive models for marine hazard prevention and environmental monitoring strategies.

## Introduction

Partial differential equations (PDEs) are fundamental mathematical tools employed across diverse scientific and engineering disciplines to model complex phenomena involving spatial and temporal variations. These equations are particularly crucial for describing wave propagation, fluid dynamics, and various physical systems where complex behaviors like shock waves, turbulence, and solitons are caused by nonlinear interactions, these equations are especially important. In this regard, the nonlinear Navier-Stokes equations are indispensable for understanding fluid motion, including complex flow patterns, turbulence, and wave breaking^1–3^. Similarly, the Korteweg-de Vries (KdV) equation has been pivotal in advancing our comprehension of nonlinear wave propagation and characterizing soliton behavior in water waves^4,5^. Beyond fundamental physics, PDEs find extensive application in environmental science and engineering. They are utilized to simulate wave propagation in atmospheric and marine systems, such as the nonlinear advection-diffusion equations that describe the dispersion of contaminants in air and water^6,7^. PDEs have a wide range of uses, from engineering applications like shock wave analysis and optical fiber research^8–10^ to environmental modeling, including climate dynamics and extreme weather events^11,12^. PDEs’ wide range of applications underscores their significance in addressing real-world challenges across a multitude of domains.

Nonlinear evolution equations (NLEEs) may be solved exactly using a variety of techniques, such as the extended Sinh-Gordon equation expansion^15^. This technique involves expanding the known solutions of the Sinh-Gordon equation to address a broader class of NLEEs. The modified extended tanh-function approach^13,14^. This method generalizes the traditional tanh-function method to obtain more comprehensive solutions. The modified extended direct algebraic method^16–18^. A systematic approach that simplifies finding solutions by transforming NLEEs into more manageable forms. The modified extended mapping method^19,20^. This method utilizes specific mappings to connect different types of solutions, enhancing the range of solvable equations. The modified Sardar Sub-Equation Method^21^. A methodology designed to extract exact solutions through the use of sub-equations derived from the original NLEEs. The extended F-Expansion method^22–24^. This method extends the F-expansion approach to formulate solutions for NLEEs more flexibly. Improved Modified Extended Tanh-Function Method, An advancement over the original method that improves computational efficiency and solution variety^25,26^. Several alternative approaches are employed, including the Homo Balance Method^27^, the Bäcklund Transformation, and Riccati-Bernoulli methods^28^, among others^31–44^. These methodologies are essential for investigating intricate physical phenomena, such as wave dynamics, fluid dynamics, and thermal interactions, while also connecting theoretical studies to practical implementations, thereby driving progress across numerous disciplines, including various branches of mathematics and physics. The exploration of NLEEs in higher-dimensional spaces continues to be an essential area of research due to its relevance in describing complex physical phenomena such as shallow water waves, plasma dynamics, and nonlinear optics. In particular, the newly proposed $(3+1)$-dimensional shallow water wave equation provides a compelling mathematical framework for modeling multidirectional wave propagation in shallow water environments. The (3+1)-dimensional shallow water wave equation, given by Eq. (1), is selected for its ability to model complex multidirectional wave interactions in shallow water environments, where high-order dispersion and nonlinear effects are significant. Unlike lower-dimensional models like the Korteweg-de Vries equation, this equation captures spatial variations in three dimensions, making it ideal for studying phenomena such as tsunamis, tidal waves, and rogue waves in coastal hydrodynamics, justifying its relevance to this study.

1 $$\begin{aligned} u_{yt} + u_{xxxy} - 3 u_{xx} u_{y} - 3 u_x u_{xy} + \lambda _1 u_{xx} + \lambda _2 u_{yy} + \lambda _3 u_{xy} + \lambda _4 u_{yz} = 0, \end{aligned}$$

where $u = u(x, y, z, t)$ is a real function and the constants $\lambda _{i} \, (i=1,2,3,4)$ govern the nonlinear and dispersive properties of the wave dynamics. A significant advancement in this field was achieved by Liu^29^, who combined symbolic computation with hybrid analytical techniques to derive exact solutions–including lump solitons, breathers, and rogue waves–for the $(3+1)$-dimensional shallow water wave equation. While Liu’s work established a foundational framework using Hirota bilinear methods and variable separation, the present study extends these results by applying IMETFM^30^ to obtain new classes of exact traveling wave solutions. Our approach systematically generates dark soliton and singular solutions, as well as hyperbolic, Jacobi elliptic, periodic, rational, and exponential solutions, thereby expanding the known solution space for this equation. Beyond deriving exact solutions, we conduct a bifurcation analysis to explore stability and dynamic transitions, offering deeper insights into wave propagation mechanisms. The efficacy of IMETFM in handling high-dimensional nonlinear systems is demonstrated, reinforcing its utility for modeling complex wave phenomena in fluid dynamics, plasma physics, and related fields. By bridging gaps in Liu’s methodology^29^, this work not only enriches the theoretical understanding of the $(3+1)$-dimensional equation but also provides practical tools for analyzing nonlinear wave interactions in physical systems.

This study is structured into six primary sections. The initial section 1 introduces the research, outlining its background and aims. The second section 2 elaborates on the IMETFM approach, while the third section 3 provides an in-depth analysis of exact solutions for the shallow water wave equation. The fourth section 4 provides graphical visualizations of the derived solutions, offering insights into their physical behaviors and wave interactions. The fifth section 5 builds upon these findings by presenting a bifurcation analysis, exploring the stability and qualitative transitions of the wave solutions in the model. The final section, Section 6, provides a conclusion, summarizing the findings and discussing the research’s implication

## IMETF method

This section delineates the core procedures of IMETFM^30^, a potent analytical tool for deriving precise solutions to (NLEEs). It effectively handles mixed-derivative terms and strong nonlinearities, yielding diverse solutions (e.g., solitons, periodic waves) with improved computational efficiency compared to traditional tanh methods. Consider a generic NLEE represented as:

2 $$\begin{aligned} P(u, u_{t}, u_{x}, u_{y}, u_{u}, u_{tt}, u_{xx}, u_{yy}, u_{zz}, \, \dots \dots ) = 0, \end{aligned}$$

where $u \equiv u(t, x, y, z)$ signifies an unknown real-valued function, and *P* is a polynomial composed of *u* and its partial derivatives w.r.t *x*, *y*, *t*, and *z*. This equation may encompass higher-order derivatives and nonlinear terms.

**Phase I:** To convert Eq. (2) into a more tractable form, we employ the traveling wave transformation:

3 $$\begin{aligned} u(x, y, z, t) = \Upsilon (\xi ), \quad \quad \xi = \textit{a}_{1} \, x + \textit{a}_{2} \, y + \textit{a}_{3} \, z + \textit{a}_{4} \, t, \end{aligned}$$

where $\textit{a}_j$ $(j = 1, 2, 3, 4)$ are constants to be determined. Substituting this transformation into Eq. (2) yields a nonlinear ODE:

4 $$\begin{aligned} G(\Upsilon , \Upsilon ', \Upsilon '', \, \dots \dots ) = 0. \end{aligned}$$

**Phase II:** We hypothesize that the solution to Eq. (4) can be expressed in the following series form:

5 $$\begin{aligned} \Upsilon (\xi ) = \sum _{i=0}^{N} r_{i} \, \mathcal {W}^{i} + \sum _{i=1}^{N} s_{i} \, \mathcal {W}^{-i}, \end{aligned}$$

where $r_{i}$ and $s_{i}$ represent constants, and $\mathcal {W}$ is a function satisfying the auxiliary differential equation:

6 $$\begin{aligned} \mathcal {W}' = \varepsilon \sqrt{c_0 + c_1 \mathcal {W} + c_2 \mathcal {W}^2 + c_3 \mathcal {W}^3 + c_4 \mathcal {W}^4}, \end{aligned}$$

with $\varepsilon = \pm 1$ and $c_j$ $(j= 0, 1, 2, 3, 4)$ denote constants. Eq. (6) provides a diverse set of fundamental solutions that facilitate the derivation of exact solutions for Eq. (2).

**Phase III:** The parameter *N* in Eq. (5) is ascertained by applying the homogeneous balance principle, which involves equating the highest-order derivative term with the highest-order nonlinear term in Eq. (4).

**Phase IV:** We construct a polynomial in $\mathcal {W}$ by using Eq. (6) and substituting Eq. (5) into Eq. (4). A system of algebraic equations is produced by gathering coefficients of like powers of $\mathcal {W}$ and setting them to zero. Computational tools like *Maple*, *MATLAB*, or *Wolfram Mathematica* are used to solve these equations and get the values of $a_{1}, a_{2}, a_{3}, a_{4}, s_{0}, r_{j},$ and $s_{j}$ (where $j = 1, 2, 3, \, \dots$). This systematic procedure leads to the perfect solutions for Eq. (2).

## Analysis of Exact Solutions

Here, we use the IMETFM to obtain exact solutions for Eq. (1). The following form of the solution is supposed:

7 $$\begin{aligned} u(x, y, z, t) = \Upsilon (\xi ), \quad \quad \xi = \alpha \, x + \beta \, y + \mu \, z + \eta \, t, \end{aligned}$$

where $\alpha$, $\beta$, $\mu$, and $\eta$ are constants representing wave numbers in the *x*, *y*, *z* directions and the temporal frequency, respectively, determining the direction and speed of the traveling wave.

Substituting the transformation in Eq. (7) into Eq. (1), we reduce it to the following nonlinear ODE:

8 $$\begin{aligned} \alpha ^3 \beta \Upsilon ^{(4)}(\xi ) + \left( \alpha ^2 \lambda _1 + \beta (\eta + \beta \lambda _2 + \alpha \lambda _3 + \mu \lambda _4) - 6 \alpha ^2 \Upsilon '(\xi ) \right) \Upsilon ''(\xi ) = 0. \end{aligned}$$

By integrating Eq. (8) once w.r.t $\xi$ and assuming the integration const. is *zero*, we get:

9 $$\begin{aligned} \alpha ^3 \beta \Upsilon '''(\xi ) + \left( \beta \eta + \alpha ^2 \lambda _1 + \beta ^2 \lambda _2 + \alpha \beta \lambda _3 + \beta \mu \lambda _4 \right) \Upsilon '(\xi ) - 3 \alpha ^2 \beta \Upsilon '(\xi )^2 = 0. \end{aligned}$$

Let

10 $$\begin{aligned} \Upsilon '(\xi )= F(\xi ). \end{aligned}$$

Substituting this into Eq. (9) yields

11 $$\begin{aligned} \alpha ^3 \beta F'(\xi ) - 3 \alpha ^2 \beta F(\xi )^2 + \left( \beta \eta + \alpha ^2 \lambda _1 + \beta ^2 \lambda _2 + \alpha \beta \lambda _3 + \beta \mu \lambda _4 \right) F(\xi ) = 0. \end{aligned}$$

To determine the form of the solution, we balance the highest-order nonlinear term $F^{2}$ with the highest-order derivative $F''$ in Eq. (11), yielding $N=2$. From (5), the resulting solution for $F(\xi )$ can be written as:

12 $$\begin{aligned} F(\xi ) = r_{0} + r_{1} \, \mathcal {W}(\xi ) + r_{2} \, \mathcal {W}(\xi )^{2} + s_{1} \, \mathcal {W}(\xi )^{-1} + s_{2} \, \mathcal {W}(\xi )^{-2}, \end{aligned}$$

where $r_{0}, r_{1}, r_{2}, s_{1}$, and $s_{2}$ are constants to be evaluated.

By incorporating $F(\xi )$ from Eq. (12) into Eq. (11) and applying Eq. (6), we extract the coefficients of $\mathcal {W}^i$ for $i \in [-4, 4]$. An algebraic set of equations is produced by equating each coefficient to zero. Solving these equations yields the perfect solutions to Eq. (1).

**Case Study 1:** When $c_0 = c_1 = c_3 = 0$, the system yields the below solution sets:

$${\textbf {[1.1]}}$$ $$r_0 = 0, \quad r_1 = 0, \quad r_2 = 2 \alpha c_4, \quad s_1 = 0, \quad s_2 = 0, \quad \lambda _3 = \frac{-\beta \eta - 4 \alpha ^3 \beta c_2 - \alpha ^2 \lambda _1 - \beta ^2 \lambda _2}{\alpha \beta }, \quad \lambda _4 = 0.$$

$${\textbf {[1.1]}}$$ $$r_0 = \frac{4 \alpha c_2}{3}, \quad r_1 = 0, \quad r_2 = 2 \alpha c_4, \quad s_1 = 0, \quad s_2 = 0, \quad \lambda _3 = \frac{-\beta \eta + 4 \alpha ^3 \beta c_2 - \alpha ^2 \lambda _1 - \beta ^2 \lambda _2}{\alpha \beta }, \quad \lambda _4 = 0.$$

Based on [1.1], the solutions to Eq. (1) are provided below:

${\textbf {[1.1.1]}}$ When $c_4 < 0$ and $c_2 > 0$, the solution is:

13 $$\begin{aligned} u_{1.1.1}(x, y, z, t) = - 2 \alpha \sqrt{c_2} \tanh \left[ \sqrt{c_2} (\alpha x + \beta y + \mu z + \eta t) \right] , \end{aligned}$$

this solution describes a dark soliton solution

${\textbf {[1.1.2]}}$ When $c_4 > 0$ and $c_2 < 0$, the solution becomes:

14 $$\begin{aligned} u_{1.1.2}(x, y, z, t) = 2 \alpha \sqrt{-c_2} \tan \left[ \sqrt{-c_2} (\alpha x + \beta y + \mu z + \eta t) \right] , \end{aligned}$$

this solution describes a singular periodic solution.

${\textbf {[1.1.3]}}$ When $c_4 > 0$ and $c_2 = 0$, the resulting solution is:

15 $$\begin{aligned} u_{1.1.3}(x, y, z, t) = - \frac{2 \alpha }{ (\alpha x + \beta y + \mu z + \eta t) }, \end{aligned}$$

this solution describes a rational solution.

Based on [1.2], the solutions to Eq. (1) are provided below:

${\textbf {[1.2.1]}}$ When $c_4 < 0$ and $c_2 > 0$, the solution is:

16 $$\begin{aligned} u_{1.2.1}(x, y, z, t) = \frac{4}{3} \alpha c_2 (\alpha x + \beta y + \mu z + \eta t) - 2 \alpha \sqrt{c_2} \tanh \left[ \sqrt{c_2} (\alpha x + \beta y + \mu z + \eta t) \right] , \end{aligned}$$

this solution describes a dark soliton solution.

${\textbf {[1.2.2]}}$ When $c_4 > 0$ and $c_2 < 0$, the solution becomes:

17 $$\begin{aligned} u_{1.2.2}(x, y, z, t) = \frac{4}{3} \alpha c_2 (\alpha x + \beta y + \mu z + \eta t) + 2 \alpha \sqrt{-c_2} \tan \left[ \sqrt{-c_2} (\alpha x + \beta y + \mu z + \eta t) \right] , \end{aligned}$$

this solution describes a singular periodic solution.

${\textbf {[1.2.3]}}$ When $c_4 > 0$ and $c_2 = 0$, the resulting solution is:

18 $$\begin{aligned} u_{1.2.3}(x, y, z, t) = - \frac{2 \alpha }{ (\alpha x + \beta y + \mu z + \eta t) }, \end{aligned}$$

this solution describes a rational solution.

**Case Study 2:** When $c_1 = c_3 = 0$, the system yields the following solution sets:

$${\textbf {[2.1]}}$$ $$r_0 = \frac{\alpha c_2}{3}, \quad r_1 = 0, \quad r_2 = 2 \alpha c_4, \quad s_1 = 0, \quad s_2 = 0, \quad \mu = \frac{-\beta \eta - 2 \alpha ^3 \beta c_2 - \alpha ^2 \lambda _1 - \beta ^2 \lambda _2 - \alpha \beta \lambda _3}{\beta \lambda _4}.$$

$${\textbf {[2.2]}}$$ $$r_0 = \alpha c_2, \quad r_1 = 0, \quad r_2 = 2 \alpha c_4, \quad s_1 = 0, \quad s_2 = 0, \quad \mu = \frac{-\beta \eta + 2 \alpha ^3 \beta c_2 - \alpha ^2 \lambda _1 - \beta ^2 \lambda _2 - \alpha \beta \lambda _3}{\beta \lambda _4}.$$

$${\textbf {[2.3]}}$$ $$r_0 = \frac{2}{3} \left( \alpha c_2 + \sqrt{\alpha ^2 c_2^2 - 3 \alpha ^2 c_0 c_4} \right) , \quad r_1 = 0, \quad r_2 = 2 \alpha c_4, \quad s_1 = 0, \quad s_2 = 0, \quad \lambda _4 = 0,$$

$$\lambda _3 = \frac{-\beta \eta + 4 \alpha ^2 \beta \sqrt{\alpha ^2 (c_2^2 - 3 c_0 c_4)} - \alpha ^2 \lambda _1 - \beta ^2 \lambda _2}{\alpha \beta }.$$

$${\textbf {[2.4]}}$$ $$r_0 = \frac{2}{3} \left( \alpha c_2 + \sqrt{\alpha ^2 c_2^2 - 3 \alpha ^2 c_0 c_4} \right) , \quad r_1 = 0, \quad r_2 = 0, \quad s_1 = 0, \quad s_2 = 2 \alpha c_0, \quad \lambda _4 = 0,$$

$$\lambda _3 = \frac{-\beta \eta + 4 \alpha ^2 \beta \sqrt{\alpha ^2 (c_2^2 - 3 c_0 c_4)} - \alpha ^2 \lambda _1 - \beta ^2 \lambda _2}{\alpha \beta }.$$

Based on [2.1], the solutions to Eq. (1) are provided below:

${\textbf {[2.1.1]}}$ If $c_4>0, c_2<0,$ and $c_0=\frac{c_2^2}{4c_4}$, then

19 $$\begin{aligned} u_{2.1.1}(x, y, z, t) = \frac{-2}{3} \alpha c_2 (\alpha x + \beta y + \mu z + \eta t) - \alpha \sqrt{-2 c_2} \tanh \left[ \sqrt{\frac{-c_2}{2}} (\alpha x + \beta y + \mu z + \eta t) \right] , \end{aligned}$$

is a dark soliton solution.

${\textbf {[2.1.2]}}$ If $c_4>0, c_2>0,$ and $c_0=\frac{c_2^2}{4c_4}$, then

20 $$\begin{aligned} u_{2.1.2}(x, y, z, t) = \frac{-2}{3} \alpha c_2 (\alpha x + \beta y + \mu z + \eta t) + \alpha \sqrt{2 c_2} \tan \left[ \sqrt{\frac{c_2}{2}} (\alpha x + \beta y + \mu z + \eta t) \right] , \end{aligned}$$

is a singular periodic solution.

Based on [2.2], the solutions to Eq. (1) are provided below:

${\textbf {[2.2.1]}}$ If $c_4>0, c_2<0,$ and $c_0=\frac{c_2^2}{4c_4}$, then

21 $$\begin{aligned} u_{2.2.1}(x, y, z, t) = - \alpha \sqrt{-2 c_2} \tanh \left[ \sqrt{\frac{-c_2}{2}} (\alpha x + \beta y + \mu z + \eta t) \right] , \end{aligned}$$

is a singular soliton solution.

${\textbf {[2.2.2]}}$ If $c_4>0, c_2>0,$ and $c_0=\frac{c_2^2}{4c_4}$, then

22 $$\begin{aligned} u_{2.2.2}(x, y, z, t) = \alpha \sqrt{2 c_2} \tan \left[ \sqrt{\frac{c_2}{2}} (\alpha x + \beta y + \mu z + \eta t) \right] , \end{aligned}$$

is a singular periodic solution.

Based on [2.3], the solutions to Eq. (1) are provided below:

${\textbf {[2.3.1]}}$ If $c_4<0, c_2>0, c_0 = \frac{{c_2}^2 m^2 (1 - m^2)}{c_4 (2 m^2 - 1)^2}$, and $\frac{1}{\sqrt{2}}<m\le 1$, then

23 $$\begin{aligned} \begin{aligned} u_{2.3.1}(x, y, z, t)&= \frac{2}{3} \alpha \sqrt{\frac{c_2}{2 m^2 - 1}} \Bigg ( (\alpha x + \beta y + \mu z + \eta t) \sqrt{\frac{c_2}{2 m^2 - 1}} \left( -(m^2 - 3 m + 1) + \sqrt{7 m^4 - 7 m^2 + 1} \right) \\&\quad - 3 \, m \, {{\varepsilon }} \left[ (\alpha x + \beta y + \mu z + \eta t) \sqrt{\frac{c_2}{2 m^2 - 1}} \right] \Bigg ), \end{aligned} \end{aligned}$$

is a Jacobi elliptic solution, where $\varepsilon \left[ (\alpha x + \beta y + \mu z + \eta t) \sqrt{\frac{c_2}{2 m^2 - 1}} \right]$ is a Jacobi epsilon function.

For $m=1$, a dark soliton-type solution is presented as follows:

24 $$\begin{aligned} u_{2.3.1a}(x, y, z, t) = \frac{2}{3} \left( 2 \alpha c_2 (\alpha x + \beta y + \mu z + \eta t) - 3 \alpha \sqrt{c_2} \tanh \left[ \sqrt{c_2} (\alpha x + \beta y + \mu z + \eta t) \right] \right) . \end{aligned}$$

${\textbf {[2.3.2]}}$ If $c_4<0, c_2>0, c_0 = \frac{{c_2}^2 (1 - m^2)}{c_4 (2 - m^2)^2}$, and $0 < m \le 1$, then

25 $$\begin{aligned} \begin{aligned} u_{2.3.2}(x, y, z, t)&= \frac{2}{3} \Bigg ( \alpha c_2 (\alpha x + \beta y + \mu z + \eta t) - \frac{3 m^2 \alpha }{c_2} \sqrt{\frac{c_2}{2 - m^2}} \, {{\varepsilon }} \left[ (\alpha x + \beta y + \mu z + \eta t) \sqrt{\frac{c_2}{2 - m^2}}\right] \\&\quad + (\alpha x + \beta y + \mu z + \eta t) \frac{\alpha c_2 \sqrt{1 - m^2 + m^4}}{m^2 - 2} \Bigg ), \end{aligned} \end{aligned}$$

is a Jacobi elliptic solution, where $\varepsilon \left[ (\alpha x + \beta y + \mu z + \eta t) \sqrt{\frac{c_2}{2 - m^2}}\right]$ is a Jacobi epsilon function.

For $m = 1$, the solutions of the dark soliton types are presented as follows:

26 $$\begin{aligned} u_{2.3.2a}(x, y, z, t) = \frac{2}{3} \left( 2 \alpha c_2 (\alpha x + \beta y + \mu z + \eta t) - \frac{3 \alpha \tanh \left[ \sqrt{c_2} (\alpha x + \beta y + \mu z + \eta t) \right] }{\sqrt{c_2}} \right) . \end{aligned}$$

${\textbf {[2.3.3]}}$ If $c_4>0, c_2<0, c_0 = \frac{{c_2}^2 m^2}{c_4 (m^2 + 1)^2}$, and $0 < m \le 1$, then

27 $$\begin{aligned} \begin{aligned} u_{2.3.3}(x, y, z, t)&= \frac{2 \, \alpha \, c_2}{3 (1 + m^2)} \Bigg ( (1 - 3 m + m^2) (\alpha x + \beta y + \mu z + \eta t) + 3 \, m \frac{ {{\varepsilon }} \left[ (\alpha x + \beta y + \mu z + \eta t) \sqrt{\frac{-c_2}{1 + m^2}}\right] }{\sqrt{\frac{-c_2}{1 + m^2}}} \\&\quad + (\alpha x + \beta y + \mu z + \eta t) \sqrt{1 - m^2 + m^4} \Bigg ), \end{aligned} \end{aligned}$$

is a Jacobi elliptic solution, where $\varepsilon \left[ (\alpha x + \beta y + \mu z + \eta t) \sqrt{\frac{-c_2}{1 + m^2}}\right]$ is a Jacobi epsilon function.

For $m=1$, a dark soliton-type solution is presented as follows:

28 $$\begin{aligned} u_{2.3.3a}(x, y, z, t) = - \alpha \sqrt{- 2 c_2} \tanh \left[ (\alpha x + \beta y + \mu z + \eta t) \sqrt{\frac{-c_2}{2}} \right] . \end{aligned}$$

Based on [2.4], the solutions to Eq. (1) are provided below:

${\textbf {[2.4.1]}}$ If $c_4>0, c_2<0, c_0 = \frac{{c_2}^2 m^2}{c_4 (m^2 + 1)^2}$, and $0 < m \le 1$, then

29 $$\begin{aligned} \begin{aligned} u_{2.4.1}(x, y, z, t)&= \frac{2}{3} \Bigg ( \frac{\alpha (\alpha x + \beta y + \mu z + \eta t) c_2}{1 + m^2} \left( -2 + m^2 - \sqrt{1 - m^2 + m^4} \right) \\&\quad - 3 \alpha \sqrt{\frac{-c_2}{1 + m^2}} \Bigg ( {{\varepsilon }} \left[ (\alpha x + \beta y + \mu z + \eta t) \sqrt{\frac{-c_2}{1 + m^2}} \right] \\&\quad + \frac{{{\,\textrm{cn}\,}}\left[ (\alpha x + \beta y + \mu z + \eta t) \sqrt{\frac{-c_2}{1 + m^2}} \right] {{\,\textrm{dn}\,}}\left[ (\alpha x + \beta y + \mu z + \eta t) \sqrt{\frac{-c_2}{1 + m^2}} \right] }{{{\,\textrm{sn}\,}}\left[ (\alpha x + \beta y + \mu z + \eta t) \sqrt{\frac{-c_2}{1 + m^2}} \right] } \Bigg ) \Bigg ), \end{aligned} \end{aligned}$$

is a Jacobi elliptic solution.

For $m=1$, a singular soliton-type solution is presented as follows:

30 $$\begin{aligned} u_{2.4.1a}(x, y, z, t) = - \alpha \sqrt{- 2 c_2} \coth \left[ (\alpha x + \beta y + \mu z + \eta t) \sqrt{\frac{-c_2}{2}} \right] . \end{aligned}$$

**Case Study 3:** When $c_0 = c_1 = c_2 = 0$, the system yields the following solution sets:

$${\textbf {[3.1]}}$$ $$r_0 = 0, \quad r_1 = \alpha c_3, \quad r_2 = 2 \alpha c_4, \quad s_1 = 0, \quad s_2 = 0, \quad \lambda _1 = 0, \quad \beta = 0.$$

Based on [3.1], the solutions to Eq. (1) are provided below:

$${\textbf {[3.1.1]}}$$ If $$c_4\ne 0$$, then

31 $$\begin{aligned} u_{3.1.1}(x, y, z, t) = -\frac{4 \alpha (\alpha x + \beta y + \mu z + \eta t) c_3^2}{(\alpha x + \beta y + \mu z + \eta t)^2 c_3^2 - 4 c_4}, \end{aligned}$$

is a rational solution.

$${\textbf {[3.1.2]}}$$ If $$c_4<0$$, then

32 $$\begin{aligned} u_{3.1.2}(x, y, z, t) = -\frac{\alpha c_3}{2 \sqrt{-c_4}} \left( 2 e^{\frac{(\alpha x + \beta y + \mu z + \eta t) c_3}{2 \sqrt{-c_4}}} + e^{\frac{(\alpha x + \beta y + \mu z + \eta t) c_3}{\sqrt{-c_4}}} \right) , \end{aligned}$$

is an exponential solution.

**Case Study 4:** When $c_0 = c_1 = 0$, the system yields the following solution sets:

$${\textbf {[4.1]}}$$ $$r_0 = 0, \quad r_1 = 0, \quad r_2 = 2 \alpha c_4, \quad s_1 = 0, \quad s_2 = 0, \quad \mu = \frac{-\beta \eta - 4 \alpha ^3 \beta c_2 - \alpha ^2 \lambda _1 - \beta ^2 \lambda _2 - \alpha \beta \lambda _3 }{\beta \lambda _4}.$$

$${\textbf {[4.2]}}$$ $$r_0 = \frac{4 \alpha c_2}{3}, \quad r_1 = 0, \quad r_2 = 2 \alpha c_4, \quad s_1 = 0, \quad s_2 = 0, \quad \mu = \frac{-\beta \eta + 4 \alpha ^3 \beta c_2 - \alpha ^2 \lambda _1 - \beta ^2 \lambda _2 - \alpha \beta \lambda _3 }{\beta \lambda _4}.$$

Based on [4.1], the solutions to Eq. (1) are provided below:

${\textbf {[4.1.1]}}$ If $c_4>0$, $c_2<0$ and $c_3 =0$, then

33 $$\begin{aligned} u_{4.1.1}(x, y, z, t) = - 2 \alpha \sqrt{-c_2} \cot \left[ \sqrt{-c_2} (\alpha x + \beta y + \mu z + \eta t) \right] , \end{aligned}$$

is a singular periodic solution.

${\textbf {[4.1.2]}}$ If $c_4>0$, $c_2>0$ and $c_3 \ne 2 \sqrt{c_2 c_4}$, then

34 $$\begin{aligned} u_{4.1.2}(x, y, z, t) = - 2 \alpha \sqrt{c_2} \coth \left[ \sqrt{c_2} (\alpha x + \beta y + \mu z + \eta t) \right] , \end{aligned}$$

is a singular soliton solution.

Based on [4.2], the solutions to Eq. (1) are provided below:

${\textbf {[4.2.1]}}$ If $c_4>0$, $c_2<0$ and $c_3 =0$, then

35 $$\begin{aligned} u_{4.2.1}(x, y, z, t) = \frac{4}{3} \alpha c_2 (\alpha x + \beta y + \mu z + \eta t) - 2 \alpha \sqrt{-c_2} \cot \left[ \sqrt{-c_2} (\alpha x + \beta y + \mu z + \eta t) \right] , \end{aligned}$$

is a singular periodic solution.

${\textbf {[4.2.2]}}$ If $c_4>0$, $c_2>0$ and $c_3 \ne 2 \sqrt{c_2 c_4}$, then

36 $$\begin{aligned} u_{4.2.2}(x, y, z, t) = \frac{4}{3} \alpha c_2 (\alpha x + \beta y + \mu z + \eta t) - 2 \alpha \sqrt{c_2} \coth \left[ \sqrt{c_2} (\alpha x + \beta y + \mu z + \eta t) \right] , \end{aligned}$$

is a singular soliton solution.

**Case Study 5:** When $c_3 = 0$, $c_4 = 0$, the system yields the following solution sets:

$${\textbf {[5.1]}}$$ $$r_0 = 0, \quad r_1 = 0, \quad r_2 = 0, \quad s_1 = \alpha c_1, \quad s_2 = \frac{\alpha c_1^2}{2 c_2}, \quad \lambda _3 = \frac{-\beta \eta - \alpha ^3 \beta c_2 - \alpha ^2 \lambda _1 - \beta ^2 \lambda _2}{\alpha \beta }, \quad \lambda _4 = 0.$$

$${\textbf {[5.2]}}$$ $$r_0 = \frac{4 \alpha c_2}{3}, \quad r_1 = 0, \quad r_2 = 0, \quad s_1 = 0, \quad s_2 = 2 \alpha c_0, \quad \lambda _3 = \frac{-\beta \eta + 4 \alpha ^3 \beta c_2 - \alpha ^2 \lambda _1 - \beta ^2 \lambda _2}{\alpha \beta }, \quad \lambda _4 = 0.$$

Based on [5.1], the solutions to Eq. (1) are provided below:

${\textbf {[5.1.1]}}$ If $c_2 > 0$ and $c_0 = \frac{c_1^2}{4 c_2}$, then

37 $$\begin{aligned} u_{5.1.1}(x, y, z, t) = \frac{2 \alpha c_1 \sqrt{c_2}}{c_1 - 2 c_2 e^{\sqrt{c_2} (\alpha x + \beta y + \mu z + \eta t)} }, \end{aligned}$$

is an exponential solution.

Based on [5.2], the solutions to Eq. (1) are provided below:

${\textbf {[5.2.1]}}$ If $c_0 > 0$, $c_2 < 0$, and $c_1 = 0$, then

38 $$\begin{aligned} u_{5.2.1}(x, y, z, t) = -2 \alpha \sqrt{-c_2} \cot \left[ \sqrt{-c_2} (\alpha x + \beta y + \mu z + \eta t) \right] + \frac{4}{3} \alpha (\alpha x + \beta y + \mu z + \eta t) c_2, \end{aligned}$$

is a singular periodic solution.

${\textbf {[5.2.2]}}$ If $c_0 > 0$, $c_2 > 0$, and $c_1 = 0$, then

39 $$\begin{aligned} u_{5.2.2}(x, y, z, t) = -2 \alpha \sqrt{c_2} \coth \left[ \sqrt{c_2} (\alpha x + \beta y + \mu z + \eta t) \right] + \frac{4}{3} \alpha (\alpha x + \beta y + \mu z + \eta t) c_2, \end{aligned}$$

is a singular soliton solution.

All solutions (Equations (13)–(39)) have been verified by substituting back into Equation 1 using computational tools like *Wolfram Mathematica*, confirming their accuracy.

## Graphical Overview

**Figure 1:**
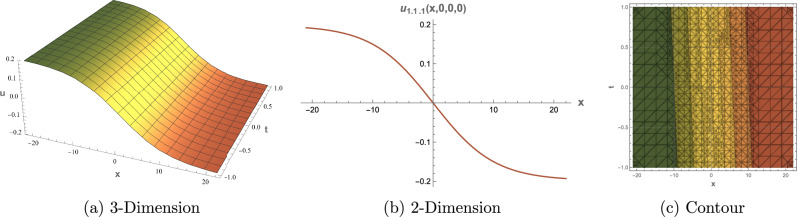
Graphical depictions of the dark soliton solution in Eq. (13). (**a**) 3D plot showing wave evolution, (**b**) 2D plot of temporal dynamics, (**c**) Contour plot of spatial amplitude distribution.

**Figure 2:**
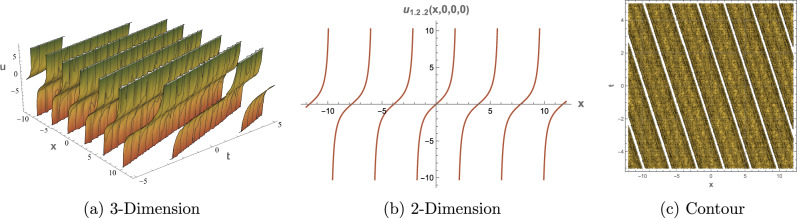
Graphical depictions of the singular periodic solution in Eq. (17). (**a**) 3D plot showing wave evolution, (**b**) 2D plot of temporal dynamics, (**c**) Contour plot of spatial amplitude distribution.

**Figure 3:**
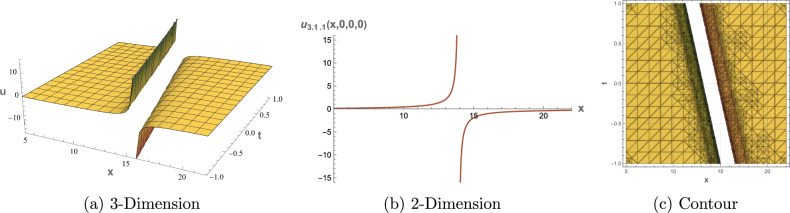
Graphical depictions of the singular soliton solution in Eq. (31). (**a**) 3D plot showing wave evolution, (**b**) 2D plot of temporal dynamics, (**c**) Contour plot of spatial amplitude distribution.

**Figure 4:**
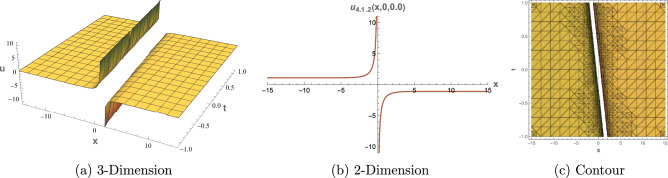
Graphical depictions of the rational solution in Eq. (34). (**a**) 3D plot showing wave evolution, (**b**) 2D plot of temporal dynamics, (**c**) Contour plot of spatial amplitude distribution.

This section presents 3D, 2D, and contour visualizations of chosen solutions to provide insight into their physical interpretations. Visualizing the solutions to the $(3+1)$-dimensional shallow water wave equation can help analysts and researchers comprehend the dynamics of wave interactions and the effects of nonlinearity and dispersion. The dimension $(3+1)$ refers to three spatial dimensions and one-time dimension, allowing for a comprehensive representation of wave behavior in a realistic setting. Common types of plots include 2D plots for showing the evolution of the wave profile over time. Contour plots for representing constant values of the wave function in the 2D space. 3D Plot To visualize how the wave travels and evolves in three dimensions. Figure 1 displays representations of the dark soliton solution of Eq. (13) with specified parameters: $\alpha = 0.70,$, $\beta = 0.80$, $\mu = 0.75$, $\eta = 0.65$ and $c_2 = 0.02$. Figure 2 provides visualizations of the singular periodic wave solution of Eq. (17) with the parameters: $\alpha = 0.90$, $\beta = 0.40$, $\mu = 0.30$, $\eta = 0.70$ and $c_2 = -0.80$. Figure 3 illustrates visualizations of the rational solution of Eq. (31) with the parameters: $\alpha = 0.40$, $\beta = 0.50$, $\mu = 0.40$, $\eta = 0.75$, $c_3 = 0.35$ and $c_4 = 0.95$. Figure 4 shows representations of the singular soliton solution of Eq. (34) with specified parameters: $\alpha = 0.50$, $\beta = 0.50$, $\mu = 0.40$, $\eta = 0.80$ and $c_2 = 1.20$. The solutions visualized in Figures 1–4 model critical physical phenomena in shallow water systems. The dark soliton (Eq. (13), Figure 1) represents stable, localized wave dips, relevant to persistent wave structures in coastal flows. The singular periodic solution (Eq.(17), Figure 2) captures oscillatory wave patterns, indicative of periodic disturbances in marine environments. The rational solution (Eq.(31), Figure 3) models divergent wave behaviors, potentially linked to wave breaking, while the singular soliton (Eq. (34), Figure 4) signifies extreme wave events, crucial for understanding coastal erosion and marine hazards, such as predicting the impact of high-amplitude waves on coastal infrastructure. These visualizations and their physical interpretations provide a robust framework for analyzing nonlinear wave interactions in hydrodynamics.

## Bifurcation Analysis

Bifurcation analysis serves as a fundamental tool for characterizing qualitative dynamical transitions in nonlinear wave systems, particularly for identifying stability thresholds and solution-type transformations in parameter space. For the $(3+1)$-dimensional shallow water wave equation, this approach becomes indispensable as it reveals critical transitions between periodic, solitary, and unstable wave regimes, and the parametric sensitivity of solution branches. We initiate our analysis from the reduced ODE (Eq. (11)) derived in Section 3, which under the conditions $\alpha \ne 0$ and $\beta \ne 0$ simplifies to the canonical form:

40 $$\begin{aligned} F''(\xi ) + \left( \frac{\eta }{\alpha ^3} + \frac{\lambda _1}{\alpha \beta } + \frac{\beta \lambda _2}{\alpha ^3} + \frac{\lambda _3}{\alpha ^2} + \frac{\mu \lambda _4}{\alpha ^3} \right) F(\xi ) - \frac{3}{\alpha } F(\xi )^2 = 0. \end{aligned}$$

For analytical clarity, we introduce the parameters:

$$\begin{aligned} \theta = \frac{\eta }{\alpha ^3}, \quad \omega = \frac{\lambda _1}{\alpha \beta } + \frac{\beta \lambda _2}{\alpha ^3} + \frac{\lambda _3}{\alpha ^2} + \frac{\mu \lambda _4}{\alpha ^3}, \end{aligned}$$

yielding the simplified form:

41 $$\begin{aligned} F''(\xi ) - \frac{3}{\alpha } F(\xi )^2 + (\theta + \omega ) F(\xi ) = 0. \end{aligned}$$

This second-order ODE transforms into a planar dynamical system through the substitution:

42 $$\begin{aligned} \frac{dF}{d\xi } = y, \quad \frac{dy}{d\xi } = -(\theta + \omega ) F(\xi ) + \frac{3}{\alpha } F(\xi )^2. \end{aligned}$$

The corresponding Hamiltonian function is:

43 $$\begin{aligned} H(F, y) = \frac{y^2}{2} + (\theta + \omega ) \frac{F^2}{2} - \frac{1}{\alpha } F^3. \end{aligned}$$

The Hamiltonian system (Eq. (43)) exhibits equilibrium points at $(0, 0)$ and $\left( \frac{\alpha (\theta + \omega )}{3}, 0 \right)$, where stability analysis reveals: for $\theta + \omega > 0$, $(0, 0)$ becomes a center (periodic waves) with eigenvalues $\sigma = \pm \sqrt{-(\theta + \omega )}$, while $\left( \frac{\alpha (\theta + \omega )}{3}, 0 \right)$ acts as a saddle with eigenvalues $\sigma = \pm \sqrt{\theta + \omega }$; for $\theta + \omega < 0$, the stabilities reverse, with a pitchfork bifurcation at $\theta + \omega = 0$ triggering transitions between periodic and soliton regimes, where Hamiltonian level curves $H(F, y) = C$ show closed orbits (periodic solutions) around centers and separatrices (solitons) near saddles, physically corresponding to wave trains versus localized pulses in the shallow water wave system.

**Figure 5:**
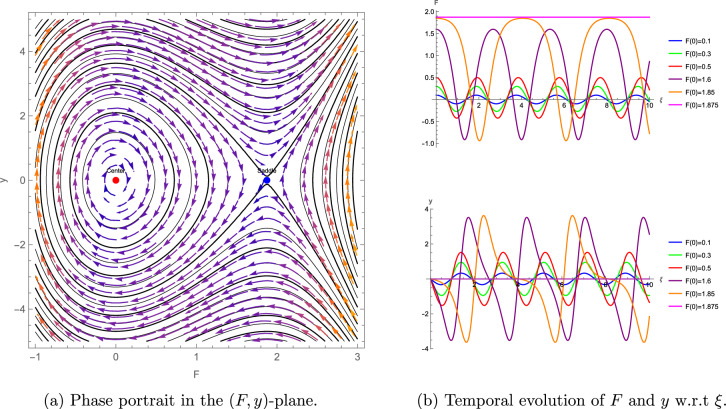
Phase portrait and temporal evolution of the planar dynamical system (42). (**a**) Shows closed orbits around the stable center at $F = 0$ and divergent trajectories near the unstable saddle at $F = 1.875$. (**b**) Depicts periodic and amplifying wave behaviors.

**Figure 6:**
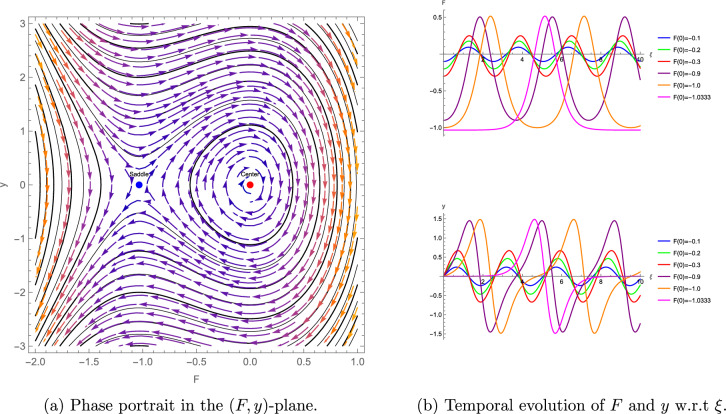
Phase portrait and temporal evolution of the planar dynamical system (42). (**a**) Shows closed orbits around the stable center at $F = 0$ and divergent trajectories near the unstable saddle at $F \approx -1.033$. (**b**) Depicts periodic and amplifying wave behaviors.

**Figure 7:**
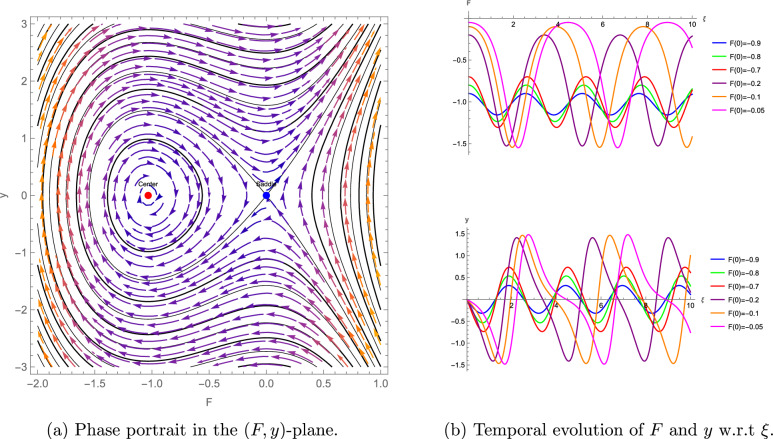
Phase portrait and temporal evolution of the planar dynamical system (42). (**a**) Shows closed orbits around the stable center at $F \approx -1.033$ and divergent trajectories near the unstable saddle at $F = 0$. (**b**) Depicts periodic and amplifying wave behaviors.

To complement the bifurcation analysis of the $(3+1)$-dimensional shallow water wave equation presented above, which elucidates the qualitative transitions and stability of wave solutions, we now turn to numerical simulations. These simulations, illustrated through phase portraits and temporal evolution plots, provide a detailed visualization of the system’s nonlinear dynamics, reinforcing the analytical findings and highlighting the practical implications of the identified wave behaviors. The numerical simulations enhance the understanding of the system’s nonlinear dynamics, as depicted in Figures 5–7. For the parameter set $\eta = 1.5$, $\alpha = 0.5$, $\beta = 1$, $\lambda _1 = -0.375$, $\lambda _2 = \lambda _3 = \lambda _4 = \mu = 0$, we compute $\theta + \omega = 11.25 > 0$, indicating a center at $F = 0$ and a saddle at $F = 1.875$, with Figure 5 (a) illustrates closed orbits at $F = 0$ and divergent trajectories at $F = 1.875$, while Figure 5 (b) shows stable and unstable temporal evolution, reflecting wave amplification. For $\eta = -0.6$, $\alpha = -0.5$, $\beta = 1$, $\lambda _1 = -0.3$, $\lambda _2 = -0.1$, $\lambda _3 = \lambda _4 = \mu = 0$, we find $\theta + \omega = 6.2 > 0$, with a center at $F = 0$ and a saddle at $F \approx -1.033$, as shown in Figure 6. For $\eta = -0.6$, $\alpha = 0.5$, $\beta = 1$, $\lambda _1 = -0.3$, $\lambda _2 = -0.1$, $\lambda _3 = \lambda _4 = \mu = 0$, we obtain $\theta + \omega = -6.2 < 0$, indicating a saddle at $F = 0$ and a center at $F \approx -1.033$, with Figure 7 (a) showing divergent trajectories at $F = 0$ and closed orbits at $F \approx -1.033$ and Figure 7 (b) shows stable and unstable temporal evolution, depicting oscillatory wave patterns.

## Conclusion

This study successfully derived a diverse set of exact traveling wave solutions for the $(3+1)$-dimensional shallow water wave equation using the Improved Modified Extended Tanh-Function Method (IMETFM). The method effectively reduced the complex high-dimensional PDE to a manageable ODE, enabling the systematic discovery of hyperbolic, trigonometric, rational, exponential, and Jacobi elliptic function solutions. These solutions capture essential nonlinear wave phenomena–including solitons, periodic waves, and singular structures–while bifurcation analysis provided critical insights into their stability and dynamic transitions. The derived solutions, including dark solitons and periodic waves, are applicable to tsunami prediction, tidal wave analysis, and coastal engineering by modeling wave amplification and interactions critical for designing early warning systems and protective infrastructure. Visualizations in 3D, 2D, and contour formats enhanced the interpretability of wave propagation and interaction dynamics. This work extends the analytical toolkit for nonlinear wave theory and offers practical value for coastal hydrodynamics and environmental hazard modeling. The results deepen our understanding of nonlinear dynamics in shallow water wave systems, demonstrating the model’s capacity to capture soliton interactions, wave amplification, and rogue wave formation. The diversity of solutions–from dark and singular solitons to periodic and rational waves–highlights the equation’s adaptability across parametric conditions. Graphical representations bridge mathematical results with physical intuition, clarifying wave evolution and spatial-temporal behavior. These visualizations underscore the method’s ability to elucidate intricate wave patterns relevant to real-world shallow water systems, such as tsunami prediction and tidal wave analysis. Comparisons with prior work, such as Liu’s study ^27^, highlight the complementary nature of IMETFM, which expands the solution space by offering new analytical forms not previously identified, thus enriching the theoretical framework for nonlinear wave studies. Future research will focus on numerical validation of solution stability under realistic shallow water conditions, including external perturbations and variable bathymetry, extending IMETFM to other high-dimensional nonlinear PDEs in fields like plasma physics and optics, and exploring integrability properties via inverse scattering transforms or machine learning approaches. These directions will strengthen the link between theoretical advances and practical applications in nonlinear wave dynamics

## Data Availability

The datasets used and/or analyzed during the current study are available from the corresponding author upon reasonable request.
